# Neural dynamics of reward probability coding: a Magnetoencephalographic study in humans

**DOI:** 10.3389/fnins.2013.00214

**Published:** 2013-11-18

**Authors:** Julie Thomas, Giovanna Vanni-Mercier, Jean-Claude Dreher

**Affiliations:** Cognitive Neuroscience Center, Reward and Decision-Making Team, CNRS, UMR 5229, Université de Lyon, Université Claude Bernard Lyon 1Lyon, France

**Keywords:** MEG, prediction-error, reward probability coding

## Abstract

Prediction of future rewards and discrepancy between actual and expected outcomes (prediction error) are crucial signals for adaptive behavior. In humans, a number of fMRI studies demonstrated that reward probability modulates these two signals in a large brain network. Yet, the spatio-temporal dynamics underlying the neural coding of reward probability remains unknown. Here, using magnetoencephalography, we investigated the neural dynamics of prediction and reward prediction error computations while subjects learned to associate cues of slot machines with monetary rewards with different probabilities. We showed that event-related magnetic fields (ERFs) arising from the visual cortex coded the expected reward value 155 ms after the cue, demonstrating that reward value signals emerge early in the visual stream. Moreover, a prediction error was reflected in ERF peaking 300 ms after the rewarded outcome and showing decreasing amplitude with higher reward probability. This prediction error signal was generated in a network including the anterior and posterior cingulate cortex. These findings pinpoint the spatio-temporal characteristics underlying reward probability coding. Together, our results provide insights into the neural dynamics underlying the ability to learn probabilistic stimuli-reward contingencies.

## Introduction

Predicting the occurrence of potentially rewarding events is a critical ability for adaptive behavior. Compelling evidence from single-unit recording in non-human primates indicate that reward probability modulates midbrain dopaminergic neurons activity both at the time of the conditioned stimuli when a prediction is made and at the time of outcome, when the discrepancy between actual and expected outcome is computed (Fiorillo et al., 2003). During appetitive classical conditioning, the phasic response of dopamine neurons increases with higher reward probability at the time of the conditioned stimulus (CS) and decreases at the time of the outcome (Fiorillo et al., 2003; Tobler et al., 2005; Kobayashi and Schultz, 2008). In humans, recent microelectrode recordings also indicate that substantia nigra neurons exhibit higher firing rates after unexpected gains than unexpected losses (Zaghloul et al., 2009). These findings support the hypotheses that midbrain dopaminergic neurons code the expected reward value at the time of the conditioned stimuli and a prediction error signal at the outcome, representing the discrepancy between anticipated and rewards effectively delivered. Building on monkey electrophysiological experiments, a number of recent fMRI studies investigated the influence of probability on reward-related brain activity (Elliott et al., 2003; Knutson et al., 2005; Abler et al., 2006; Dreher et al., 2006; Preuschoff et al., 2006; Tobler et al., 2007). Although most of these studies focused on reward probability coding in the ventral striatum, the representations of reward expectation and of reward prediction errors are not confined to subcortical regions and are also found in cingulate, prefrontal, and intra-parietal cortices (Platt and Glimcher, 1999; Fletcher et al., 2001; Ridderinkhof et al., 2004; Sugrue et al., 2004; Dreher et al., 2006).

Much less is known concerning the representations of reward properties in early visual structures. However, recent theoretical proposals and empirical findings in humans support the view that reward properties, such as reward timing (Shuler and Bear, 2006) and prior reward history of stimuli (Serences, 2008; Gavornik et al., 2009) may be coded in areas of the visual system, including V1. Moreover, a recent fMRI study in monkeys showed that dopaminergic signals can modulate visual cortical activity, directly demonstrating that reward help to regulate selective plasticity within the visual representation of reward predicting stimuli (Arsenault et al., 2013). These studies challenge the common view that only after initial processing of low-level stimulus features in the visual cortex are higher order cortical areas engaged to process the significance of visual input and its predictive value.

The vast majority of neuroimaging studies performed in humans used fMRI to investigate the influence of reward probability on the brain. However, because of the low temporal resolution of fMRI, it has not been possible to investigate the precise timing of reward probability coding and their spatio-temporal characteristics during conditioning tasks using designs with short CS-outcome period as those used in animal experiments, nor to separate events temporally close within these tasks (Fiorillo et al., 2003; Dreher et al., 2006; Tobler et al., 2007). Other approaches using techniques with high temporal resolution, such as EEG, have focused on the neural mechanisms of feedback evaluation when subjects evaluate outcomes of their actions and use these evaluations to guide decision-making (Gehring and Willoughby, 2002; Holroyd et al., 2004; Cohen et al., 2007; Christie and Tata, 2009). However, these types of tasks may not involve the same processes as those required in classical conditioning.

Here, we report a new MEG study investigating the spatio-temporal neural dynamics of prediction and reward prediction errors computations by focusing on the timing of responses to predictive cues and rewarded/unrewarded outcomes when humans learned probabilistic associations of visual cues of slot machines with monetary rewards. We characterized the effects of reward probability on evoked magnetic fields occurring during the computations underlying reward prediction and reward prediction error.

## Methods

### Participants

Twelve right-handed subjects (seven males) participated in this MEG experiment (mean age ± *SD* 23.08 ± 2.23 years). They were all university students, and were free of psychiatric and neurological problems as well as of drug abuse and history of pathological gambling. Four subjects (3 males) were excluded from the data analysis because of large head movements (>5 mm). All subjects gave their written, informed consent to participate in this study, which was approved by the local ethics committee. The subjects were paid for their participation, and earned extra money in proportion to their gains during the experiment.

### Experimental procedure

**Figure 1:**
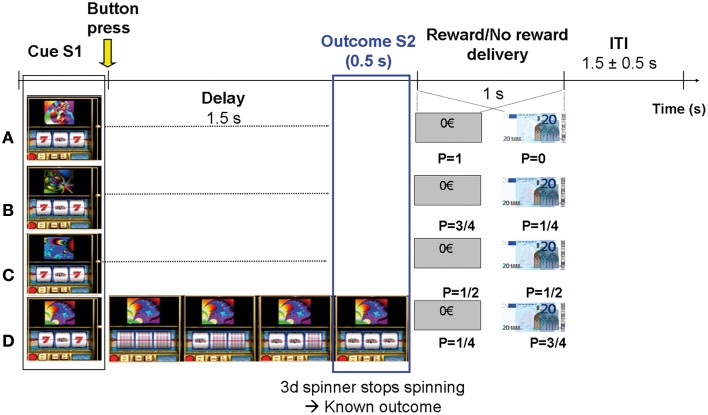
**Experimental paradigm**. Subjects estimated the reward probability of 4 types of slot machines that varied with respect to monetary reward probabilities *P* (0, 0.25, 0.5, and 0.75). The slot machines could be discriminated by specific fractal images on top of them. Trials were self-paced and were composed of 4 distinct phases: (1) Slot machine presentation (S1): subjects pressed one of two response-keys to estimate whether the slot machine frequently delivered 20€ or not, over all the past trials; (2) Delay period (1.5 s): Subject's key press triggered 3 spinners to roll around and to successively stop every 0.5 s during 0.5 s; (3) Outcome S2 (lasting 0.5 s): the 3rd spinner stopped spinning revealing the trial outcome (i.e., fully informing the subject on subsequent reward or no reward delivery). Only two configurations were possible at the time the third spinner stopped: “bar, bar, seven” (no reward) or “bar, bar, bar” (reward); (4) Reward/No reward delivery (1 s): picture of 20€ bill or rectangle with 0€ written inside.

Subjects were presented with 10 runs of 4 blocks with the same elementary structure (Figure 1). In each block, one single slot machine was presented on a computer screen during 20 consecutive trials (*ITI* = 1.5 ± 0.5 s). Each slot machine was made visually unique by displaying a particular fractal image on top of it. In each run, 4 types of slot machines were presented in random order and unbeknownst to the subjects were attached to 4 reward probabilities (*P* = 0; 0.25; 0.5; 0.75). A total of 10 * 4 = 40 different slot machines were presented. The probability of each slot machine was exact and reached at the end of each block. The paradigm is similar to the one used in a previous intra-cranial recording study (Vanni-Mercier et al., 2009). Briefly, the subjects' task was to estimate at each trial the reward probability of each slot machine at the time of its presentation, based upon all the previous outcomes of the slot machine until this trial (i.e., estimate of *cumulative* probability since the first trial). To do so, subjects had to press one of two response-buttons: one button indicating that, overall, the slot machine had a “high winning probability” and the other button indicating that overall, the slot machine had a “low winning probability.” Thus, the task was *not* to predict whether the current slot machine would be rewarded or not rewarded on the current trial. Subjects were told that their current estimate had no influence on subsequent reward occurrence. During the task, subjects received no feed-back relative to their correct/incorrect estimation of the winning probability of the slot machine. Finally, at the end of each block, they were asked to classify the slot machine on a scale from 0 to 4 according to their global estimate of reward delivery over the block. The experimental paradigm was implemented with the Presentation software (http://nbs.neuro-bs.com/presentation).

### MEG recordings and MRI anatomical scans

MEG recordings were carried out in a magnetically shielded room with a helmet shaped 275 channels gradiometer whole-head system (OMEGA; CTF Systems, VSM Medtech, Vancouver, BC, Canada). The subjects were comfortably seated upright, instructed to maintain their heads motionless and to refrain from blinking. Head motions were restricted by using a head stabilizer bladder. Between runs, subjects could have a short rest with eye blinking allowed, but were asked to stay still. Visual stimuli were projected on a translucent screen positioned 1.5 m from the subject. The subject's head position relative to MEG sensors was recorded at the beginning and end of each run using three anatomical fiducials (coils fixed at the nasion and at left and right preauricular points). These coils were also used to co-register the MEG sensor positions with the individual anatomical MRI. Subject's head position was readjusted between runs to maintain the same position all along the experiment. Subjects with head movements larger than 5 mm were excluded from the study. The mean head movement of the subjects included in the analysis was 3.5 ± 0.8 mm.

Anatomical MRI scans were obtained for each subject using a high resolution T1-weighted sequence (magnetization prepared gradient echo sequence, MP-RAGE: 176 1.0 mm sagital slices; *FOV* = 256 mm, *NEX* = 1, *TR* = 1970 ms, *TE* = 3.93 ms; matrix = 256 × 256; *TI* = 1100 ms, bandwidth = 130Hz/pixel for 256 pixels in-plane resolution = 1 mm^3^).

MEG signals were sampled at 600 Hz with a 300 Hz cut-off filter and stored together with electro-oculogram (EOG), electrocardiogram (ECG) signals, and digital markers of specific events for subsequent off-line analysis. These markers included: 4 markers at the cue (slot machine appearance: S1) corresponding to the 4 reward probabilities of the slot machines (P0, 0.25, 0.5, and 0.75), 2 markers at the subject's behavioral responses, and 7 markers at the outcome (i.e., when the 3rd spinner stops spinning: S2), defined according to the 7 possible outcomes (3 rewarded slot machines, 3 unrewarded slot machines, and one with only unrewarded outcomes).

### Behavioral data analysis

**Figure 2:**
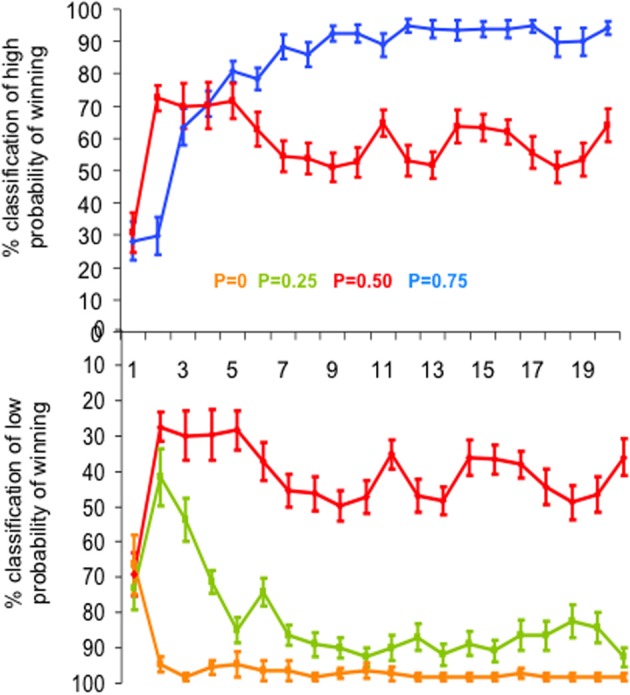
**Behavioral performance**. Mean learning curves averaged across subjects, expressed as the mean percentage of “high probability of winning” **(top)** and “low probability of winning” **(bottom)** estimations of the four slot machines, as a function of trial rank. Vertical bars represent standard errors of the mean. Each slot machine is color coded (with reward probability from 0 to 0.75). Note that the subjects' task was simply to estimate at each trial the reward probability of each slot machine at the time of its presentation, based upon all the previous outcomes of the slot machine until this trial (i.e., estimate of cumulative probability since the first trial). To do so, subjects had to press one of two response-buttons: “high winning probability” and “low winning probability.” In particular, the estimation of the slot machine with *P* = 0.5 of winning reached the learning criterion (i.e., >80% correct estimations) after the 7th trial (estimations oscillating around 50% as “high” or “low” probability of winning).

We computed the percentage of correct estimations of the reward probability for each slot machine as a function of trial rank (from 1 to 20) averaged over subjects and runs (Figure 2). Estimations were defined as correct when subjects classified as “low winning” the slot machines with low reward probabilities (*P* = 0 and *P* = 0.25) and as “high winning” the slot machines with high reward probability (*P* = 0.75). Since the slot machine with reward probability *P* = 0.5 had neither “low” nor “high” winning probability and the choice was binary, the percent of 50% estimates of “high winning,” or symmetrically of “low winning” probability, corresponded to the correct estimate of winning probability for this slot machine.

For the probabilities P0, P0.25, and P0.75, the trial rank when learning occurred (learning criterion) was defined as the trial rank with at least 80% of correct responses and for which the percent of correct estimation did not decrease below this limit for the remaining trials. For the probability *P* = 0.5, the trial rank when learning occurred was defined as the trial rank with approximately 50% of the responses being either “high” or “low winning” probability and then oscillating around this value for the remaining trials. Moreover, results from subjects' estimation of the slot machines for each of the 20 successive presentations of a single type of slot machine within runs were compared to their classification made at the end of each block. We also analyzed the response time (RT) as a function of reward probability.

### MEG data processing

MEG signals were converted to a 3rd order synthetic gradient, decimated at 300 Hz, Direct Current (DC) corrected and band-pass filtered between 0.1 and 30 Hz. They were then processed with the software package for electrophysiological analyses (ELAN-Pack) developed at the INSERM U821 laboratory (Lyon, France; http://u821.lyon.inserm.fr/index_en.php). Trials with eye blinks or muscle activity were discarded after visual inspection, as well as cardiac artifacts, using the program DataHandler developed at the CNRS-LENA UPR 640 laboratory (Paris, France, http://cogimage.dsi.cnrs.fr/index.htm) (~10% of rejection in total). Signals were averaged with respect to the markers at S1 and S2 using epochs of 3500 ms (−1500 to 2000 ms from the markers) with a 1000 ms baseline correction. Baseline activity was defined as the average activity during the ITI.

### MEG data analysis

#### Event-related magnetic fields

For each reward probability, we ensured the statistical significance of the ERFs observed at the cue presentation (S1) and at the outcome (S2) compared to the baseline with a Wilcoxon test performed on epochs of 3500 ms, with a moving time-window of 20 ms shifted by 2 ms step.

Next, we examined the relationship between ERFs peak amplitudes and reward probability at the group level at S1 and S2, using an ANOVA with reward probability as independent factor. *Post-hoc* comparisons were then performed using Tukey's HSD tests to further assess the significant differences between ERFs peak amplitudes as a function of probability. In addition, we determined the mean onset latencies, peak latencies, and durations of the ERFs time-locked to S1 and S2.

#### Sources reconstruction at S1

**Figure 3:**
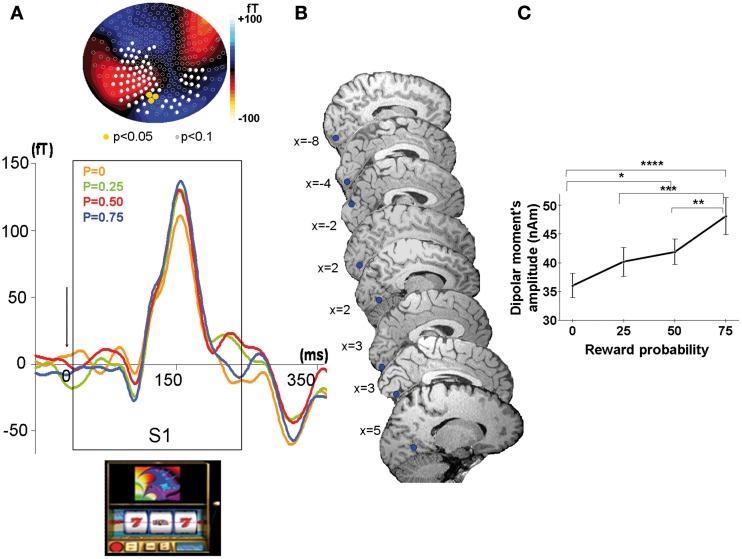
**MEG response sensitive to the conditioned stimulus at the time of the cue (S1). (A)** Top: Scalp topography of the M150. Evoked related magnetic fields maps averaged across all participants showing a linear increase with reward probability at the time of the peak amplitude (=155 ms) after cue presentation (S1). Color scale indicates the intensity of the magnetic field. Color dots indicate occipital sensors (sensors MLO11, MLO12, MLO22) where the ERFs showed a significant modulation by reward probability (Yellow dots: *P* < 0.05; white dots: *P* < 0.1). Bottom: Grand average MEG waveform on one selected occipital sensor in the left hemisphere (sensor MLO11) for the presentation of the slot machines (cue at S1), linearly modulated by reward probability (*P* = 0: yellow, *P* = 0.25: green, *P* = 0.5: red, *P* = 0.75: blue). **(B)** Source localization showing highly reproducible dipoles on saggital slices in each subject (*n* = 8). **(C)** Linear increase with reward probability of the dipolar moment's amplitudes of the first dipole explaining the activity elicited by the cue. Stars represent statistical differences found with a Tukey *post-hoc* between dipolar moment's amplitudes ^*^p < 0.05; ^**^p < 0.005; ^***^p < 0.0005; ^****^p < 0.00005. Vertical bars represent standard errors of the mean.

Sources reconstruction of averaged ERFs was performed according to two different methods at S1. We used the dipolefit analysis (DipoleFit, CTF Systems, Inc., Vancouver, BC, Canada) for the ERFs observed at S1 because at the time of the cue the scalp distribution of ERFs was clearly dipolar (Figure 3A). An advantage of this technique is that dipole pairs can be fitted to each individual dataset separately. The signal time-window used for dipole localization was 90–150 ms after S1, during the rising phase of the ERFs up to its point of maximal amplitude, because this rising phase is considered to reflect the primary source activation of the signal. No *a priori* hypothesis was made concerning the localization of dipoles necessary to explain the MEG activity recorded at the sensors level. For each source given by the default settings of the analysis software, the residual variance was calculated and the potential source was accepted if the residual variance was less than 15%. We added dipoles in a parsimonious way to reach this threshold. For each subject, 2 or 3 dipoles explained the signal with 85% of goodness-of-fit, but only the first dipole was observed in each individual subject at similar location in the visual cortex. Therefore, we considered this first dipole as the most plausible and its localization was performed in each subject using the BrainVoyager software (http://www.brainvoyager.com). The final criterion for the acceptance of the defined potential dipole was its physiological plausibility (location in gray matter and amplitude <250 nA m). Finally, we performed the analysis of dipole moments amplitudes as a function of reward probability at the group level with a multifactorial ANOVA.

#### Sources reconstruction at S2

For the sources reconstruction of the ERFs observed at S2, we used the Synthetic Aperture Magnetometry (SAM) methodology (CTF Systems, Inc., Vancouver, BC, Canada) because in this case, the MEG signals were more complex (multipeaked) and their scalp distribution was clearly multipolar. SAM is an adaptive spatial filtering algorithm or “beamformer.” The beamformer is a linear combination of the signals recorded on all sensors, optimized such that the signal estimated at a point is unattenuated while remote correlated interfering sources are suppressed. It links each voxel in the brain with the MEG sensors by constructing an optimum spatial filter for that location (Van Veen et al., 1997; Robinson and Vrba, 1999). This spatial filter is a set of weights and the source strength at the target location is computed as the weighted sum of all the MEG sensors. The algorithms of the SAM method are reported in (Hillebrand and Barnes, 2005). SAM examines the changes in signal power in a certain frequency band between two conditions for each volume element. We applied SAM to the 0.1–40 Hz frequency band to provide a statistical measure of the difference in the signal power of the two experimental conditions over the same time-window. We compared the two extreme reward probabilities at S2. That is, we focused on searching the sources showing a difference in power between *P* = 0.25 and *P* = 0.75 at S2 for rewarded trials (reflecting a prediction error). The time-window chosen for the analysis of the ERFs included the first ERF peak, in accordance with the significant effects observed on the sensors that is, from 0 to 300 ms. SAM analysis was applied on the whole brain volume with a 2.5 mm voxel resolution. The true *t*-test value was calculated at each voxel with a Jackknife approach. The Jackknife method enables accurate determination of trial-by-trial variability, while integrating the multiple covariance matrices over all but one trial. For each of these covariance matrices, SAM computes the source power on a voxel by voxel basis.

These statistical SAM data were superimposed on individual MRI data of each subject and statistical parametric maps representing significant voxels as color volumes were generated in each subject. We then performed a co-registration of the individual subjects' statistical maps on a “mean” brain obtained from all subjects. At the individual level, *p*-values < 0.05 were considered as significant (i.e., *t* > 2, Jackknife test). At the group level, the anatomical source locations were reported (Tables 1, 2) and displayed (Figure 5) if at least half of the subjects reached a statistical significance of *t* > 2 (Jackknife test).

**Table 1:** **Sources localizations of the first individual dipoles found at S1 reported in Talairach space, and their corresponding ellipsoid of confidence volume (*p* ≥ 95%)**.

**Anatomical structures**	**Reward value**			**Volume error (cm3)**
	***x***	***y***	***z***	
CS	−8	−87	9	0.72
CS	−4	−87	0	0.001
CS/Cuneus	−2	−86	16	0.035
CS/Cuneus	2	−87	21	0.01
CS	1	−86	−3	0.24
CS	3	−73	21	0.69
CS	3	76	19	0.59
CS	5	−65	19	0.77

Abbreviation: CS, Calcarine Sulcus.

**Table 2:** **Sources localizations of ERFs found at S2 for all the subjects, in Talairach space**.

**Anatomical structures**	**Reward prediction error**		
	***x***	***y***	***z***
ACC	5	43	19
Post. ACC	5	−36	48
Precuneus	5	−2	54
SMG	−26	−38	44

Abbreviations: ACC, Anterior Cingulate Cortex; SMG, Supramarginal gyrus.

## Results

### Behavioral results

#### Estimation of reward probability

A multifactorial ANOVA performed on the percent of correct estimates of the probability of winning (low likelihood of winning for P0 and P0.25, high likelihood of winning for P0.75 and 50% of each alternative for P0.5) showed that both reward probability (P) and trial rank (R) influenced the percentage of correct estimations [*F*_*P*(3, 1460)_ = 220.2, *p* < 0.0001; *F*_*R*(19, 1460)_ = 16.7, *p* < 0.0001]. The trial rank at which learning occurred depended on reward probability [interaction between trial rank and probability: *F*_*R* * *P*(57, 1460)_ = 6.9, *p* < 0.001]: that is, reward probability P0 reached the learning criterion (i.e., >80% correct estimations) after the 2nd trial, while the reward probabilities P0.25 and 0.75 reached the learning criterion after the 5th (respectively 84.9 and 80.7% correct estimations). The reward probability P0.5 reached the learning criterion after the 7^th^ trial (estimations oscillating around 50% as “high” or “low” probability of winning) (Figure 2).

In addition, the global estimate of reward delivery performed over each block confirmed that subjects learned the reward probability of each slot machine: they made 97.5% of correct estimations for reward probability *P* = 0; 77.5% for *P* = 0.25; 68.8% for *P* = 0.75 and 82.5% for *P* = 0.5.

Together, these behavioral results indicate that learning of cue-outcome contingencies was performed rapidly for each type of slot machine (even for the slot machine with *P* = 0.5). Thus, because the learning criterion was reached rapidly (in 2–7 trials) the effect of learning on MEG signals could not be studied and the MEG signals were analyzed after averaging all trials.

### Response times (RTs)

RTs were analyzed using a One-Way ANOVA with reward probability (P) as independent factor. No main effect of the probability of the slot machines on RT [*F*_(3, 8696)_ =1.4, *p* = 0.238] was observed. The mean RT ± SEM for all reward probabilities and trials was: 673.3 ± 28.2 ms.

### MEG signals

#### MEG-evoked responses at the sensor level

***Modulation of ERFs observed at the cue (S1) by reward probability.*** In each subject, strong ERFs emerged at left occipital sensors (MLO 11, 12, 22) around 90 ms after S1 (appearance of the slot machine), peaking at 155 ms ± 13 ms and lasting up to 260 ms ± 11.4 ms after the cue onset. We therefore analyzed the ERFs averaged across all subjects. For each type of slot machine (i.e., reward probability), this emerging signal was significantly different from baseline during a time window varying from 35 to 265 ms around the maximal amplitude (Wilcoxon tests, *p*-values varying from <0.0001 to <0.02 at the different sensors). At all the occipital sensors showing the ERFs at S1, there was a main effect of reward probability on the peak amplitude of these ERFs (110 to 137 fT) [ANOVA with probability as independent factor: MLO11: *F*_(3, 5580)_ = 6.3, *p* < 0.0005; MLO12: *F*_(3, 5580)_ = 2.8, *p* < 0. 05, MLO22: *F*_(3, 5580)_ = 2.6, *p* < 0.05]. Moreover, a test of linearity (Spearman) revealed that these ERFs increased linearly with reward probability at all these sensors, being minimal for P0.25 and maximal for P0.75 (*p* < 0.05) (Figure 3A).

**Figure 4:**
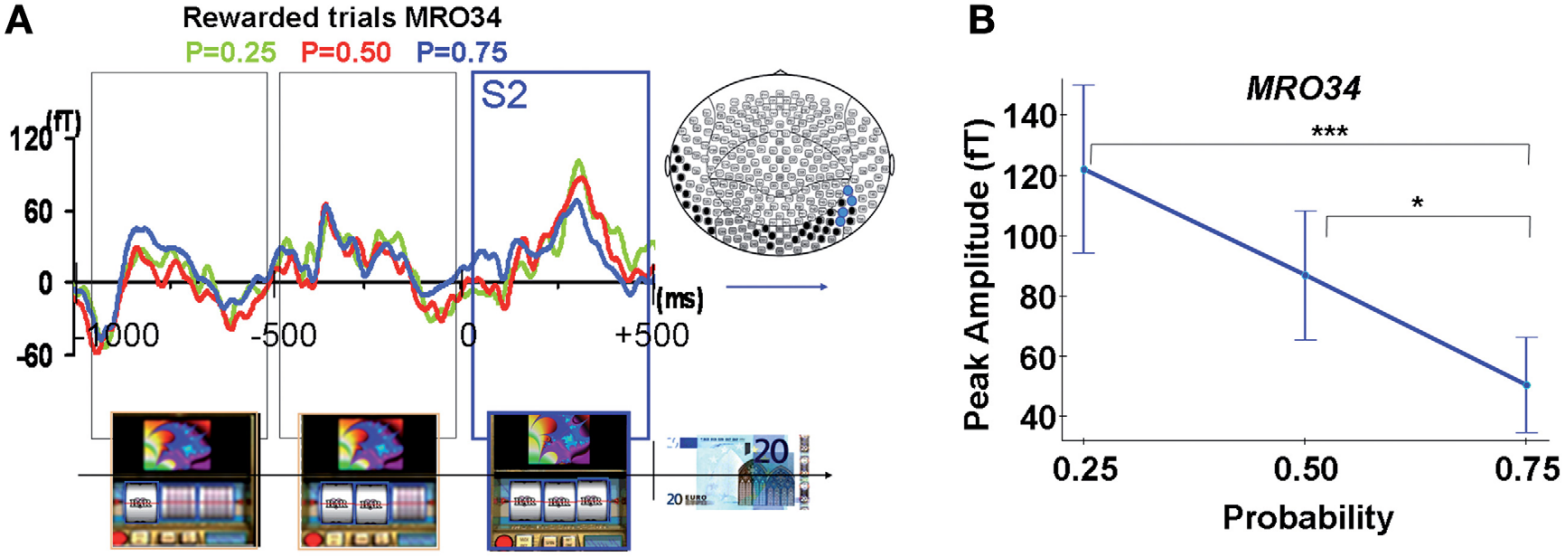
**MEG response occurring during spinner's rotations and at the time of the outcome S2. (A)** Averaged signal from a representative selected occipital site (sensor MRO34), during spinners' rotation and showing a linear modulation with reward probability when the third spinner stopped (S2) for rewarded trials (Reward probability *P* = 0.25: green; *P* = 0.5: red; *P* = 0.75: blue). Color dots on the map's brain indicate sensors where there was a statistical difference between peaks amplitudes for rewarded trials as a function of reward probability (blue dots: *P* < 0.05, black dots: *P* < 0.1). **(B)** ERF's peak amplitudes from a representative selected occipital site (sensor MRO34) modulated by reward probability for unrewarded outcomes. Stars represent statistical differences (Tukey *post-hoc* between different probabilities. ^*^P < 0.05; ^***^P < 0.0005). Vertical bars represent standard errors of the mean.

***Modulation of ERFs observed at S2 by reward probability.*** Early complex ERFs emerged over occipital and temporal areas 110 ± 11.4 ms after each successive stop of the three spinners of the slot machines, peaking 300 ± 16.5 ms after and lasting 450 ± 13.2 ms. Only after the third spinner stopped, giving full information about upcoming outcome (S2), was the peak amplitude of these ERFs (68–99 fT), observed at occipital and temporal sensors (MRO34, MRT15, MRT26, MRT27), modulated by reward probability (Figures 4A,B). This signal was significantly different from baseline for each reward probability during a time window varying from 20 to 450 ms around the maximal amplitude for rewarded trials (Wilcoxon tests, *p*-values varying from <0.0001 to <0.043). ANOVAs performed at the group level showed a main effect of probability. That is, reward probability modulated ERFs' peak amplitudes for rewarded trials [sensors MRO34: *F*_Probability(2, 2173)_ = 10.8, *p* < 0.00005; MRT 15: *F*_Probability(2, 2173)_ = 5.2, *p* < 0.05; MRT 26: *F*_probability(2, 2173)_ = 7.1, *p* < 0.005; MRT27: *F*_Probability(2, 2173)_ = 3.6, *p* < 0.05]. Moreover, tests of linearity (Spearman) between ERFs' peak amplitudes and reward probability at all these sites were significant, decreasing linearly from *P* = 0.25 to *P* = 0.75 for rewarded trials (*p* < 0.05).

### Sources reconstructions of ERFs

#### Sources reconstruction at S1

The localization of the dipole source of the ERFs observed at S1 was consistently assigned to the calcarine sulcus for 6 of the 8 subjects and to the cuneus/calcarine junction for the remaining 2 subjects (Figure 3B and Table 1). The relationship between the amplitudes of these dipolar moments averaged across subjects and reward probability monotonically increased with reward probability, being minimal for *P* = 0.25 and maximal for *P* = 0.75. An ANOVA at the group level, with reward probability as independent factor showed a main effect of reward probability on dipolar moment amplitude's [*F*_(3, 5580)_ = 14.8, *p* < 5.10^−6^] (Figure 3C). A subsequent test of linearity (Spearman) between dipolar moments amplitudes and reward probability revealed that these dipolar moments amplitudes increased linearly with reward probability (*p* < 0.05) (Figure 3C).

#### Sources reconstruction at S2

**Figure 5:**
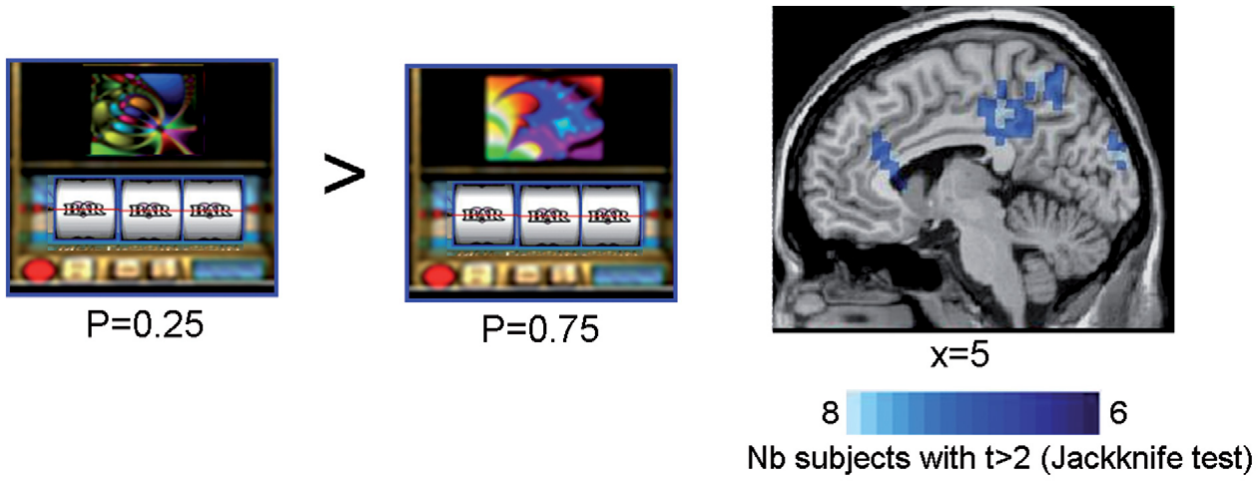
**Synthetic Aperture Magnetometry activation maps reflecting reward prediction error at S2**. SAM activation maps showing higher activity with lower reward probability for rewarded trials at the time the third spinner stopped (S2). Rewarded trials at *P* = 0.25 *vs.* rewarded trials at *P* = 0.75 induced a variation in the power of the sources in the anterior cingulate cortices, the precuneus, and the supramarginal gyrus. Color maps indicate the number of subjects showing a difference in source power for lower reward probability for rewarded trials at the outcome S2 with *t* > 2 (Jackknife test).

In all subjects but one, SAM activation maps identified a set of sources of the ERFs observed at the outcome S2 for rewarded trials as a function of reward probability. When comparing the sources powers between the lowest (*P* = 0.25) and the highest (*P* = 0.75) rewarded probability, we observed that the anterior and posterior cingulate cortices, the precuneus and the supramarginal gyrus (Brodmann's area 40) were more activated by the lowest (*P* = 0.25) than the highest (*P* = 0.75) rewarded probability (Figure 5 and Table 2) (Jackknife tests, *t* > 2).

## Discussion

This study used a probabilistic reward task to characterize the spatio-temporal dynamics of cerebral activity underlying reward processing using MEG. Two important results emerge from the present study: (1) reward value is coded early (155 ms) after the cue in the visual cortex; (2) prediction error is coded 300 ms after the outcome S2 (i.e., when the third spinner stops, fully informing subjects on subsequent reward/no reward delivery) in a network involving the anterior and posterior cingulate cortices. Moreover, reward probability modulates both ERFs coding reward prediction at the cue and prediction error at the outcome.

## Early reward value coding at the time of the cue

We found early evoked-related magnetic fields (ERFs) arising around 90 ms and peaking 155 ms after the cue presentation over the occipital cortex (M150) (Figure 3A). Dipole source localization showed that this early response observed at mid-occipital site was most probably generated in primary visual areas (calcarine sulcus) and to a lesser extent in secondary visual areas (Figure 3B). The amplitude of dipolar moments corresponding to these sources increased linearly with reward probability, indicating that visual areas code the reward value of the cue very early (Figure 3C). The ERFs observed at the cue are unlikely to represent the response to low level stimulus attributes of the fractal displayed on the slot machine because each of these fractals was associated with a distinct reward probability in each block, and the ERFs associated to each probability represents the mean response to all the different fractals having the same reward probability averaged over the ten runs.

Several studies have documented value-based modulations in different areas of the visual system. In rodents, primary visual cortex neurons code the timing of reward delivery (Shuler and Bear, 2006) and in humans, the visual primary cortex was found to be modulated by prior reward history in a two-choice task (Serences, 2008), showing that primary visual areas are not exclusively involved in processing low-level stimuli properties. Our results extend these findings in two important ways: first, by revealing that reward value is coded early in the primary visual cortex during a conditioning paradigm, and second, by showing that reward probability effectively modulates this early signal in a parametric way. The representation of reward value in early visual areas could be useful to increase attention to the cues having higher reward probability and to bias decisions for high value cues. Consistent with our finding, a recent EEG/MEG study investigating reward anticipation coding reported differential activity over midline electrodes and parieto-occipital sensors (Doñamayor et al., 2012). Differences between non-reward and reward predicting cues were localized in the cuneus, also interpreted as a modulation by reward information during early visual processing.

One open question concerns whether reward probability is computed locally in the visual cortex or whether this information is received from afferent areas coding reward value even earlier than 150 ms, either directly from midbrain dopaminergic neurons or indirectly from the basal ganglia or other cortical areas. The early latency of visual cortex response to reward value (emerging at 90 ms and peaking at 150 ms) observed in the current study is compatible with direct inputs from the substantia nigra/VTA neurons that show short-latency responses (~50–100 ms) to reward delivery (Schultz, 1998) and with a recent monkey fMRI study showing that dopaminergic signals modulate visual cortex activity (Arsenault et al., 2013). In contrast, this latency would have been slower if the response resulted from polysynaptic sub-cortical or cortical routes (Thorpe and Fabre-Thorpe, 2001).

## Reward prediction error coding at the outcome

Although ERFs emerged over occipito-temporal sensors 90 ms after each successive stop of the three spinners of the slot machines, only after the third spinner stopped at the outcome S2—fully informing the subject on subsequent reward/no reward delivery—were these ERFs modulated by reward probability. The ERFs observed during the first two time-windows most probably reflect a motion illusion effect (Figure 4A), in line with the known functional specialization motion-selective units in area MT and the important role of the temporal cortex in motion perception (Kawakami et al., 2000; Senior et al., 2000; Schoenfeld et al., 2003; Sofue et al., 2003; Tanaka et al., 2007).

At the outcome, ERFs peaked around 300 ms over temporo-occipital sensors. The mean peak amplitudes of these ERFs followed the properties of a reward prediction error signal (Figures 4B, 5). That is, these peak amplitudes decreased linearly with reward probability for rewarded outcomes. Our results show that coding of the prediction error occurs rapidly when the third spinner stops, and before the actual reward/no reward delivery. Moreover, we disentangle the prediction error signal from the signal occurring at the time of reward delivery, often confounded in fMRI studies because of its limited temporal resolution (Behrens et al., 2008; D'Ardenne et al., 2008).

Source localization of the ERFs observed with the prediction error identified sources such as the ACC and the posterior cingulate cortex, as well as the parietal cortex. The modulation of the peak amplitude of the ERFs by reward probability observed at sites generated by ACC/posterior cingulate cortices may reflect that this signal is conveyed by dopaminergic neurons, known to exhibit discharges dependent upon reward probability (Fiorillo et al., 2003).

The ERFs observed in the current study at the outcome, peaking around 300 ms after the third spinner stopped is functionally reminiscent of both the M300, the magnetic counterpart to the electric P300 (Kessler et al., 2005) and the magnetic equivalent of the feedback-related negative activity (mFRN). The FRN and the feedback-related P300 are two ERPs components which have been of particular interest when a feedback stimulus indicates a win or a loss to a player in a gambling game. The FRN is a fronto-central negative difference in the ERP following losses or error feedback compared to wins or positive feedback, peaking around 300 ms (Holroyd et al., 2003; Yasuda et al., 2004; Hajcak et al., 2005a,b), which may reflect a neural response to prediction errors during reinforcement learning (Holroyd and Coles, 2002; Nieuwenhuis et al., 2004). The P300, a parietally distributed ERP component, is sensitive to the processing of infrequent or unexpected events (Nieuwenhuis et al., 2005; Verleger et al., 2005; Barcelo et al., 2007). Larger P300s are elicited by negative feedback when participants thought they made a correct response, by positive feedback when participants thought they made an incorrect response (Horst et al., 1980). Our results are in accordance with previous reports of reduced amplitude of either the FRN (Holroyd et al., 2009, 2011; Potts et al., 2011; Doñamayor et al., 2012) or the ensuing P300 (Hajcak et al., 2005a,b) following expected compared to unexpected non-rewarding outcomes.

As the prediction error signal found in the current study, both the FRN and P300 evoked potentials are sensitive to stimulus probability (Nieuwenhuis et al., 2005; Verleger et al., 2005; Barcelo et al., 2007; Cohen et al., 2007; Mars et al., 2008). However, our conditioning paradigm markedly differs from decision making paradigms classically used to observe feedback-related signals (Falkenstein et al., 1991; Nieuwenhuis et al., 2004; Cohen et al., 2007; Bellebaum and Daum, 2008; Christie and Tata, 2009; Cavanagh et al., 2010), making systematic functional comparisons difficult. For the same reason, it is difficult to compare our results with those of a recent MEG study using a risk aversion paradigm in which subjects had to choose between two risky gambles leading to potential losses and gains (Talmi et al., 2012). The analysis of this study, limited to the sensor level, reported a signal that resembled the FRN emerging around 320 ms after outcome. Although this study argued that the presence of a condition with losses may strengthen conclusions regarding reward prediction error, potential losses can also induce counterfactual effects (Mellers et al., 1997), which is a problem when the same reference point (i.e., no gain) is not included in each gamble (Breiter et al., 2001). Indeed, the emotional response to the outcome of a gamble depends not only on the obtained outcome but also on its alternatives (Mellers et al., 1997). Thus, the neural mechanisms of feedback evaluation after risky gambles likely involve different processes as those engaged in a simple classical conditioning paradigm. Yet, all these paradigms indicate to the subjects the difference between prediction and actual outcomes and participate to different forms of reinforcement learning and evaluation of outcomes of decisions to guide reward-seeking behavior.

Further studies are needed to dissociate the different roles of feedback in risky decision making, in classical and instrumental conditioning and in social decision making, involving both common and specific neural mechanisms. For example, concerning the counterfactual effect mentioned above, recent fMRI and MEG studies investigated brain responses involved in the feeling of regret and disappointment by manipulating the feedback participants saw after making a decision to play certain gambles: full-feedback (regret) vs. partial-feedback (disappointment: when only the outcome from chosen gamble is presented) (Coricelli and Rustichini, 2010; Giorgetta et al., 2013). Another question relates to whether the FRN and ACC activities express salience prediction errors rather than reward prediction errors, as suggested by recent EEG and fMRI data using different types of rewards and punishments (Metereau and Dreher, 2013; Talmi et al., 2013). Studies manipulating different rewards and punishments are needed to clarify this question (but see Dreher, 2013; Sescousse et al., 2013).

## Conclusion

The current study identified the spatio-temporal characteristics underlying reward probability coding in the human brain. It provides evidence that the brain computes separate signals, at a sub-second time scale, in successive brain areas along a temporal sequence when expecting a potential reward. First, processing of reward value at the cue takes place at an early stage in visual cortical areas, and then the anterior and posterior cingulate cortices, together with the parietal cortex compute a prediction error signal sensitive to reward probability at the time of the outcome. Together, these results suggest that these signals are necessary when expecting a reward and when learning probabilistic stimuli-outcome associations. Our findings provide important insights into the neurobiological mechanisms underlying the ability to code reward probability, to predict upcoming rewards and to detect changes between these predictions and rewards effectively delivered.

### Conflict of interest statement

The authors declare that the research was conducted in the absence of any commercial or financial relationships that could be construed as a potential conflict of interest.
